# High-intensity interval training improves respiratory and cardiovascular adjustments before and after initiation of exercise

**DOI:** 10.3389/fphys.2024.1227316

**Published:** 2024-03-11

**Authors:** Go Ito, Marina Feeley, Toru Sawai, Hideomi Nakata, Shingo Otsuki, Hidehiro Nakahara, Tadayoshi Miyamoto

**Affiliations:** ^1^ Graduate School of Human Environment, Osaka Sangyo University, Daito City, Osaka, Japan; ^2^ Department of Sport and Health Sciences, Faculty of Sport and Health Sciences, Osaka Sangyo University, Daito City, Osaka, Japan; ^3^ Graduate School of Health Sciences, Morinomiya University of Medical Sciences, Osaka City, Osaka, Japan

**Keywords:** constant-load exercise, ramp-load exercise, maximal oxygen uptake, heart rate, blood pressure, maximal exercise performance

## Abstract

**Purpose:** High-intensity interval training (HIIT) may induce training-specific physiological adaptations such as improved respiratory and cardiovascular adjustments before and after the onset of high-intensity exercise, leading to improved exercise performance during high-intensity exercise. The present study investigated the effects of HIIT on time-dependent cardiorespiratory adjustment during maximal exercise and before and after initiation of high-intensity exercise, as well as on maximal exercise performance.

**Methods:** 21 healthy male college students were randomly assigned to HIIT group (n = 11) or control group (n = 10). HIIT group performed training on a cycle ergometer once a week for 8 weeks. The training consisted of three bouts of exercise at 95% maximal work rate (WR_max_) until exhaustion. Before and after the HIIT program, dynamic cardiorespiratory function was investigated by ramp and step exercise tests, and HIIT-induced cardiac morphological changes were assessed using echocardiography.

**Results:** HIIT significantly improved not only maximal oxygen uptake and minute ventilation, but also maximal heart rate (HR), systolic blood pressure (SBP), and time to exhaustion in both exercise tests (*p* < 0.05). Time-dependent increases in minute ventilation (V_E_) and HR before and at the start of exercise were significantly enhanced after HIIT. During high-intensity exercise, there was a strong correlation between percent change (from before to after HIIT program) in time to exhaustion and percent change in HR_max_ (r = 0.932, *p* < 0.001). Furthermore, HIIT-induced cardiac morphological changes such as ventricular wall hypertrophy was observed (*p* < 0.001).

**Conclusion:** We have demonstrated that HIIT at 95% WR_max_ induces training-specific adaptations such as improved cardiorespiratory adjustments, not only during maximal exercise but also before and after the onset of high-intensity exercise, improvement of exercise performance mainly associated with circulatory systems.

## Introduction

High-intensity interval training (HIIT) has been reported to improve maximal aerobic capacity and exercise performance, achieve higher training effects with shorter training periods, and produce maximal cardiorespiratory improvement similar to continuous training, making it widely used in Europe and globally (Tabata et al., 1996; Rognmo et al., 2004; Wisløff et al., 2007; Monks et al., 2017; Kercher et al., 2023). Matsuo et al. (2014) examined the effects of HIIT at different training intensities five times a week for 8 weeks, and reported comparable increases in aerobic exercise capacity and cardiac morphological changes regardless of training intensity and contents. Stepto et al. (1999) reported that HIIT at markedly different training intensities (85% vs. 175% maximal power output) performed twice a week for 3 weeks improved post-training maximal power and 40-km time trial performance to similar extent. Recently, similar results were reported by Litleskare et al. (2020) who compared training-specific adaptations to 8 weeks of continuous moderate-intensity training with sprint interval training, and reported that both training modalities achieved similar improvements in several performance measures including maximal oxygen uptake (V_O2max_). Our previous study also showed that once-weekly HIIT significantly improved V_O2max_ and cardiorespiratory function during submaximal exercise, induced cardiac morphological adaptations, and produced a training effect similar to other HIIT protocols performed several times a week (Nakahara et al., 2015). Therefore, many HIIT studies have reported significant adaptive changes in the cardiorespiratory system, including decreased resting systolic blood pressure (SBP) and myocardial hypertrophy, similar to continuous training, regardless of training intensity or frequency. Moreover, HIIT has been found to be effective in improving high blood pressure and cardiorespiratory function not only in adults and the elderly but also in children and adolescents (Domaradzki et al., 2022; Popowczak et al., 2022). Thus, investigating these parameters both at rest and during maximum exercise is crucial for understanding the adaptation of the circulatory function to HIIT. However, the effects of HIIT on time-dependent cardiorespiratory responses during ramp exercise and before and after the initiation of high-intensity step exercise, and the relationship between adaptive changes in the cardiorespiratory system and improved exercise performance have not been investigated. In a recent report, we have shown that heart rate (HR) response before the initiation of high-intensity step exercise is altered in an intensity-dependent manner by neural drives from higher centers, and is closely related to faster HR response to exercise and determines maximal exercise performance (Miyamoto et al., 2022). This rapid HR adjustment, both before and after beginning the exercise, helps enhance the oxygen (O_2_) supply to the muscles that are active. Consequently, this enables individuals to rapidly adapt to the energy requirement of constant load submaximal work, leading to a reduced O_2_ deficit. Thus, we hypothesize that HIIT may induce training-specific physiological adaptations such as improved respiratory and cardiovascular adjustments not only during maximal exercise, but also before and after the onset of high-intensity exercise, leading to improved exercise performance. Furthermore, the combination of HR acceleration and enhanced myocardial contractility, a result of cardiac hypertrophy from HIIT-induced cardiovascular adaptations, not only improves cardiac function during maximal exertion but also influences the SBP response in a time-dependent manner during high-intensity exercise. This effect allows for maintaining elevated blood pressure longer after high-intensity exercise begins, facilitating extended durations of such exercise. Moreover, this enhanced cardiac and circulatory response significantly supports the circulatory system’s ability to supply oxygen continuously to active muscles from the start of exercise, thereby boosting overall performance during high-intensity activities. To prove the above hypothesis, the present study examined the effects of HIIT on intensity-dependent dynamic cardiorespiratory response before and after initiation of step and ramp exercise tests, and investigated the relationship between exercise training-induced adaptive cardiorespiratory changes and improved exercise performance.

## Materials and methods

### Subjects

21 healthy male college students with no cardiovascular risk factors participated in the study (Table 1). The subjects’ exercise habits involved engaging in light exercise and sports once or twice a week. They were healthy male college students with normal fitness levels, although they were not considered well-trained athletes. Subjects were randomly assigned to one of the following two groups: HIIT group (n = 11, age: 21 ± 1, weight: 84 ± 17 kg, height: 174 ± 6 cm, BMI: 28 ± 6) and control group (n = 10, age: 21 ± 2, weight: 80 ± 24 kg, height: 170 ± 8 cm, BMI: 27 ± 6). Pre- and post-intervention physical properties were not different between the HIIT and control groups. All subjects were informed about the experimental procedures, potential risks and discomfort, and signed an informed consent form. The present study was approved by the Human Subjects Committee of Morinomiya University of Medical Sciences (No. 2018-056) and Osaka Sangyo University (No. 2021-21).

**TABLE 1:** Comparison of resting cardiorespiratory variables before and after intervention in the HIIT and control groups.

	HIIT group		Control group		Mixed effect ANOVA (*p*-value)		
	(n = 11)		(n = 10)		Main effect		Interaction effect
	Pre	Post	Pre	Post	Group (G)	Training (T)	G × T
Rest							
V_O2_ (mL/min)	402 ± 96	425 ± 108	498 ± 103	508 ± 113	0.069	0.125	0.942
V_E_ (L/min)	13 ± 2	13 ± 3	15 ± 2	15 ± 3	0.016	0.681	0.928
V_T_ (mL)	944 ± 392	882 ± 262	1,203 ± 294	1,206 ± 338	0.085	0.774	0.185
RR (breaths/min)	15 ± 4	16 ± 2	13 ± 3	13 ± 3	0.386	0.411	0.788
HR (beats/min)	80 ± 11	82 ± 13	76 ± 14	75 ± 12	0.173	0.725	0.189
SBP (mmHg)	124 ± 10	116 ± 11**	123 ± 7	122 ± 6	0.513	0.002	0.026
DBP (mmHg)	81 ± 8	76 ± 10	79 ± 9	83 ± 8	0.344	0.782	0.176
MBP (mmHg)	93 ± 7	86 ± 12	94 ± 8	96 ± 7	0.073	0.281	0.097

Values are presented as mean ± SD. HIIT, high-intensity interval training; V_O2_, oxygen uptake; V_E_, minute ventilation; V_T_, tidal volume; RR, respiratory rate; HR, heart rate; SBP, systolic blood pressure; DBP, diastolic blood pressure; MBP, mean blood pressure; Pre, before intervention; Post, after intervention. **p < 0.01 vs. before HIIT, program.

### Experimental protocols and procedures

The intervention period was 8 weeks, during which the HIIT group underwent one session of exercise training per week, while the control did not undergo exercise training. Before and after the intervention period, all subjects underwent ramp exercise test and step exercise test (high-intensity constant-load exercise) for investigation of cardiorespiratory function. For 24 h preceding the day of an exercise test, the subjects were instructed to avoid strenuous exercise and to continue their usual diet, but to avoid food with a high salt content. Food, alcohol, and caffeine were prohibited 4 h before each test. Ramp exercise test was conducted at 2 weeks before the intervention period, and 1 week after the intervention period. Step exercise test was conducted at 1 week before and 2 weeks after the intervention period. Left ventricular adaptations were assessed by echocardiography before and after the intervention period. Echocardiographic examination was conducted before a ramp exercise test.

### Exercise training protocol

**FIGURE 1:**
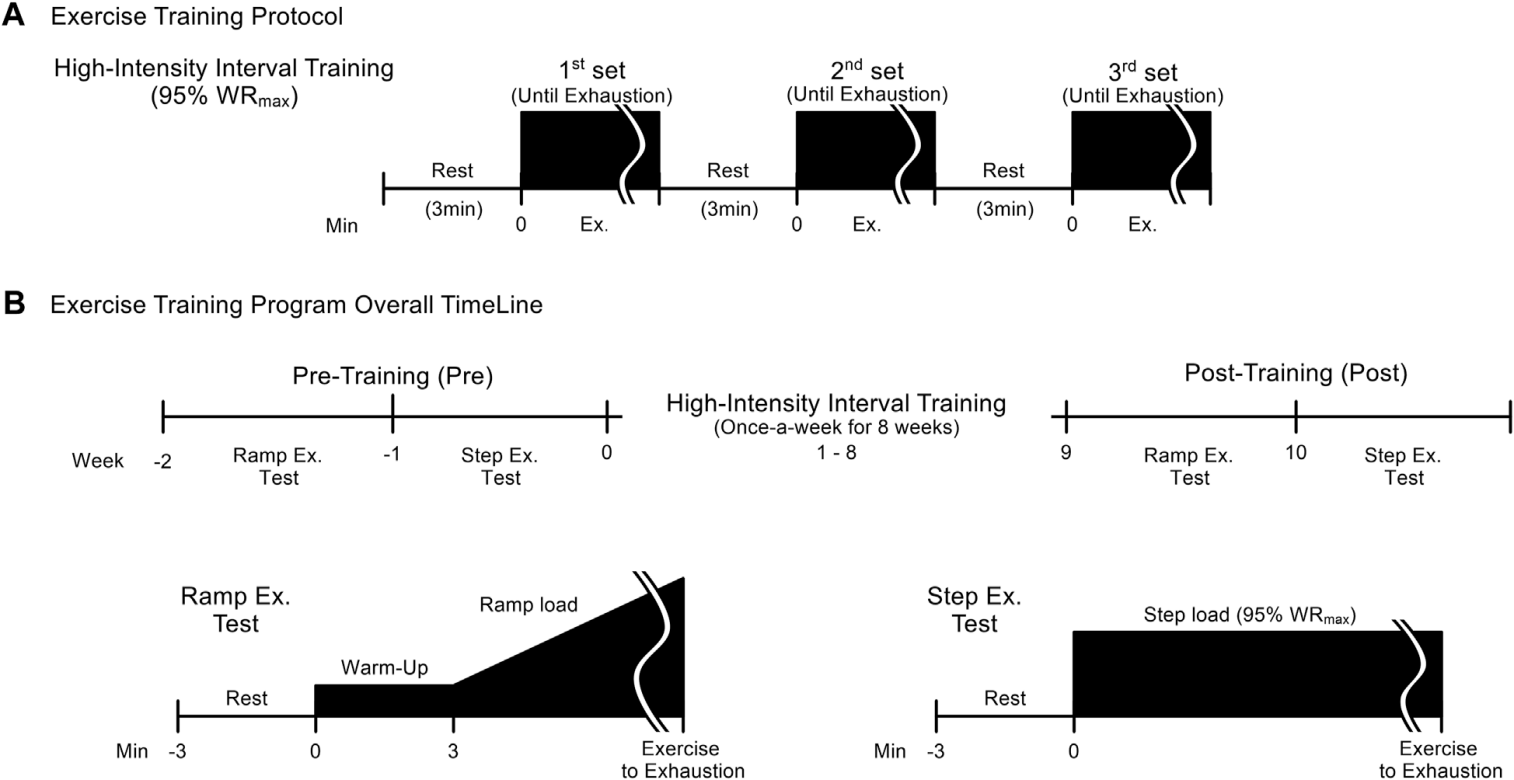
High-intensity interval training (HIIT) protocol **(A)** and overall timeline of HIIT and pre- and post-training tests **(B)**. The HIIT protocol consists of 3 sets of step exercise at an intensity of 95% maximal work rate performed until exhaustion, with 3 minutes of rest between sets, once a week for 8 weeks. Before and after the HIIT program, two exercise tests: a ramp (week -2 and week 9, respectively) and a step exercise test (week -1 and week 10) are conducted. The test methods are shown in **(B)**.

The HIIT group conducted high-intensity interval training at a frequency of one session per week for 8 weeks (Figure 1A). The exercise training program involved bicycle ergometer exercise as described in our previous report (Nakahara et al., 2015; Nakahara et al., 2020). Each training session consisted of three bouts of exercise at 95% maximal work rate (WR_max_) until exhaustion. The 3-min recovery period between training bouts consisted of 2 min of active recovery at 0 W, which provides an appropriate balance between intracellular restitution and maintenance of high oxygen uptake kinetics (Seiler and Hetlelid, 2005), followed by 1 min of rest (Figure 1A). During exercise training, the pedaling speed was maintained at around 60 rpm until the subject could no longer maintain a pedaling rate above 50 rpm despite strong verbal encouragement. At each training session, all subjects were interviewed to ensure that they had not changed their routine activities apart from the HIIT program. The control group did not perform any exercise other than routine activities during the intervention period.

### Ramp exercise test

In each subject, V_O2max_ was assessed as the maximal aerobic capacity by a ramp exercise test using a computer-controlled bicycle ergometer (AEROBIKE 75XL, Combi wellness Co., Tokyo). The work rate was set initially at 20 W for 3 min as warm-up, and increased by 20 W every minute (Figure 1B). During exercise, the pedaling rate was maintained at approximately 60 rpm, until the subject could no longer maintain a pedaling rate above 50 rpm despite strong verbal encouragement. The criteria for the achievement of V_O2max_ were a plateau in O_2_ uptake (V_O2_) despite increased work rate, and a respiratory exchange ratio above 1.10. Other cardiorespiratory functions were also measured together with V_O2max_. Physical properties and maximal cardiorespiratory capacity of the two experimental groups are given in Tables 1, 2. The WR_max_ achieved by each subject during the ramp exercise test was defined as 100% WR_max_, and based on this value, relative WR for the subsequent tests was determined for each subject.

**TABLE 2:** Comparison of maximal cardiorespiratory variables and exercise performance during the ramp and step exercise tests conducted before and after intervention in the HIIT and control groups.

	HIIT group		Control group		Mixed effect ANOVA (*p*-value)		
	(n = 11)		(n = 10)		Main effect		Interaction effect
	Pre	Post	Pre	Post	Group (G)	Training (T)	G × T
Ramp exercise protocol							
V_O2max_ (mL/min)	3,158 ± 646	3,518 ± 437**	3,134 ± 470	3,087 ± 365	0.306	0.033	0.008
V_Emax_ (L/min)	124 ± 25	153 ± 23***	121 ± 11	121 ± 12	0.039	0.002	0.002
V_Tmax_ (mL)	2,761 ± 788	2,868 ± 387	2,822 ± 551	3,018 ± 424	0.661	0.17	0.677
RR_max_ (breaths/min)	58 ± 9	66 ± 10**	53 ± 10	51 ± 11	0.037	0.105	0.001
HR_max_ (beats/min)	190 ± 8	196 ± 6*	186 ± 14	185 ± 11	0.115	0.14	0.035
SBP_max_ (mmHg)	189 ± 15	206 ± 18**	198 ± 19	198 ± 18	0.962	0.012	0.017
DBP_max_ (mmHg)	103 ± 14	100 ± 12	106 ± 10	102 ± 15	0.611	0.292	0.923
MBP_max_ (mmHg)	128 ± 14	130 ± 10	133 ± 9	130 ± 16	0.654	0.971	0.399
WR_max_ (watt)	294 ± 40	330 ± 42***	269 ± 20	270 ± 24	0.008	0.001	0.001
Step exercise protocol							
V_O2max_ (mL/min)	3,239 ± 502	3,560 ± 587	3,036 ± 501	3,122 ± 440	0.17	0.008	0.106
V_Emax_ (L/min)	130 ± 32	151 ± 19**	122 ± 20	123 ± 14	0.073	0.017	0.028
V_Tmax_ (mL)	2,753 ± 547	3,019 ± 563	2,723 ± 449	2,926 ± 576	0.79	0.019	0.732
RR_max_ (breaths/min)	55 ± 16	60 ± 10	53 ± 12	51 ± 11	0.299	0.496	0.114
HR_max_ (beats/min)	174 ± 9	184 ± 8**	173 ± 12	175 ± 15	0.33	0.003	0.049
SBP_max_ (mmHg)	179 ± 22	197 ± 19**	196 ± 19	194 ± 19	0.451	0.015	0.003
DBP_max_ (mmHg)	94 ± 11	98 ± 8	95 ± 21	94 ± 14	0.858	0.608	0.478
MBP_max_ (mmHg)	117 ± 12	127 ± 8	124 ± 21	124 ± 14	0.774	0.068	0.068
Exhaustion time (sec)	130 ± 16	249 ± 44***	137 ± 30	151 ± 29	0.001	<0.001	<0.001

Values are presented as mean ± SD. HIIT, high-intensity interval training; Pre, before intervention; Post, after intervention; V_O2max_, maximal oxygen uptake; V_Emax_, maximal minute ventilation; V_Tmax_, maximal tidal volume; RR_max_, maximal respiratory rate; HR_max_, maximal heart rate; SBP_max_, maximal systolic blood pressure; DBP_max_, maximal diastolic blood pressure; MBP_max_, maximal mean blood pressure; WR_max_, maximal work rate during the ramp exercise; Exhaustion time, time to exhaustion in the step exercise test. *p < 0.05, **p < 0.01, ***p < 0.001 vs. before HIIT, program.

### Step exercise test

Cardiorespiratory function and maximal intense exercise performance were measured during step exercise test using a bicycle ergometer. The work rate for the step exercise test was set at 95% WR_max_ for all subjects, both before and after intervention (Figure 1B). During exercise, the pedaling rate was maintained at approximately 60 rpm. The time to exhaustion was defined as the interval from exercise onset until the subject could no longer maintain a pedaling frequency above 50 rpm despite strong verbal encouragement.

### Experimental apparatus and measurements

Respiratory and metabolic data during the exercise tests were recorded using an automatic breath-by-breath gas analyzing system (ARCO2000-MET; Arcosystem, Chiba, Japan) consisting of a differential pressure transducer, sampling tube, filter, suction pump, and mass spectrometer. We recorded expired flow, CO_2_ and O_2_ concentrations; and derived tidal volume (V_T_), respiratory rate (RR), minute ventilation (V_E_), V_O2_, and CO_2_ output (V_CO2_) from the digitized data. The gas analyzers were calibrated before and after each test. HR was monitored via a three-lead electrocardiogram (BSM-7201; Nihon Kohden, Tokyo, Japan), and beat-to-beat HR was recorded continuously using a personal computer in on-line mode at a sampling rate of 200 Hz during each test. Blood pressure (BP) was monitored using an automatic indirect manometer (EBP-300, Minato Med. Sci. Co. Osaka, Japan) worn on the participant’s left arm while seated on the cycle ergometer. Mean blood pressure (MBP) was calculated from measured SBP and diastolic blood pressure (DBP) using the formula: (SBP - DBP)/3 + DBP. During each test, HR, O_2_, CO_2_ and flow signals were recorded continuously at 200 Hz. BP were recorded continuously every 30 s. M-mode echocardiography was conducted using an SSD 6500 ultrasound device (Aloka, Tokyo, Japan) and a sector probe, with the subject supine. M-mode left ventricular measurements were derived from the parasternal long-axis view, and the septal and posterior wall thicknesses of the left ventricle and the dimensions of the ventricle cavities were measured in triplicate according to the recommendations of the American Society of Echocardiography (Sahn et al., 1978). Left ventricular mass was calculated using Devereux’s formula (Devereux et al., 1986). Left ventricular volume was computed using the Teichholz rule. All analyses were performed by one investigator.

### Data analysis

For resting data in both groups, mean cardiorespiratory responses of each subject were calculated from the measurements averaged over the first minute of the 3-min resting period before each exercise test. For each exercise test performed until reaching exhaustion, measurements of cardiorespiratory variables were averaged over 10 s. The maximal 10-s averaged values of cardiovascular variables were used as the subject’s maximal cardiorespiratory function data. During the step exercise test, the dynamic cardiorespiratory responses before the start of exercise (−40 to 0 s) and after the start of exercise (0–40 s) were calculated using data averaged over 10 s. The BPs measured during the ramp and step exercise tests were averaged every 1 min, both before and after the start of exercise. Time to exhaustion was defined as the interval from onset of exercise until the subject could no longer maintain a pedaling frequency above 50 rpm despite strong verbal encouragement. Each echocardiographic measurement was the mean of three continuously repeated echocardiographic measurements. Missing data on blood pressure in the post-training HIIT group (ramp and step exercise test, n = 10 and n = 11, respectively) and the control group (ramp and step exercise test, n = 8 and n = 8, respectively) have been replaced by the group’s mean response at that time point for statistical analysis.

### Statistical analysis

T-test was used to compare the mean values of two groups. When the objective was to discern the main effects and potential interactions between the variables of ‘Group’ and “Training”, a two-way mixed-effect ANOVA for repeated measures was employed, utilizing the mean data of individual subjects. Further, to accommodate intra-individual comparisons and to unravel the main effects of “Group”, “Training”, as well as “Time”—along with any interactions amongst these factors—a three-way mixed-effect ANOVA was conducted. This encompassed an additional within-subject factor, “Time”, segmented into four discrete intervals (5 s, 15 s, 25 s, and 35 s). The model was meticulously crafted to evaluate the fixed effects of “Group” (HIIT vs. CONTROL) and “Training” (Pre vs. Post), while also accounting for the random effect attributed to individual participants. This facilitated the accommodation of the repeated measures design intrinsic to our study. The inclusion of the “Time” element permitted an in-depth analysis of respiratory and circulatory responses within each training session, thereby capturing the nuanced dynamics of cardiorespiratory responsiveness to the HIIT protocol. We integrated an interaction term involving “Group”, “Training”, and “Time” within our model to ascertain if the HR response to the training regimen exhibited variability not only between the groups but also across the different time intervals within the training sessions. In instances where significant interaction effects surfaced, the analysis was meticulously refined through the application of the Tukey Honest Significant Difference (HSD) *post hoc* test. This pivotal step allowed for a granular examination of the specific mean differences spanning multiple comparisons, particularly within the HIIT group, across the diverse time intervals between pre- and post-training sessions. For the evaluation of the V_O2_–HR relationship during the ramp exercise test, we resorted to multiple regression analysis. This facilitated the computation and subsequent comparison of the slope before and after the intervention. Furthermore, the Pearson product-moment correlation coefficient (r) analysis was leveraged to probe into the relationship between the percent change in HRmax and the percent change in time to exhaustion during the step exercise test, with the percent change computed as [(post-intervention value–pre-intervention value)/pre-intervention value × 100%]. All data are articulated as mean ± SD unless stated otherwise. For all statistical analyses conducted, we upheld a criterion for statistical significance at *p* < 0.05.

## Results

Table 1 compared the resting cardiorespiratory variables between the HIIT and control groups before and after intervention. Resting SBP in the HIIT group decreased significantly (−6%, *p* = 0.004) before and after the intervention (Table 1). Two-way ANOVA revealed a significant group × training interaction and the main effect of training for this variable. However, other pre- and post-intervention resting cardiorespiratory variables were not different between the HIIT and control groups.

**FIGURE 2:**
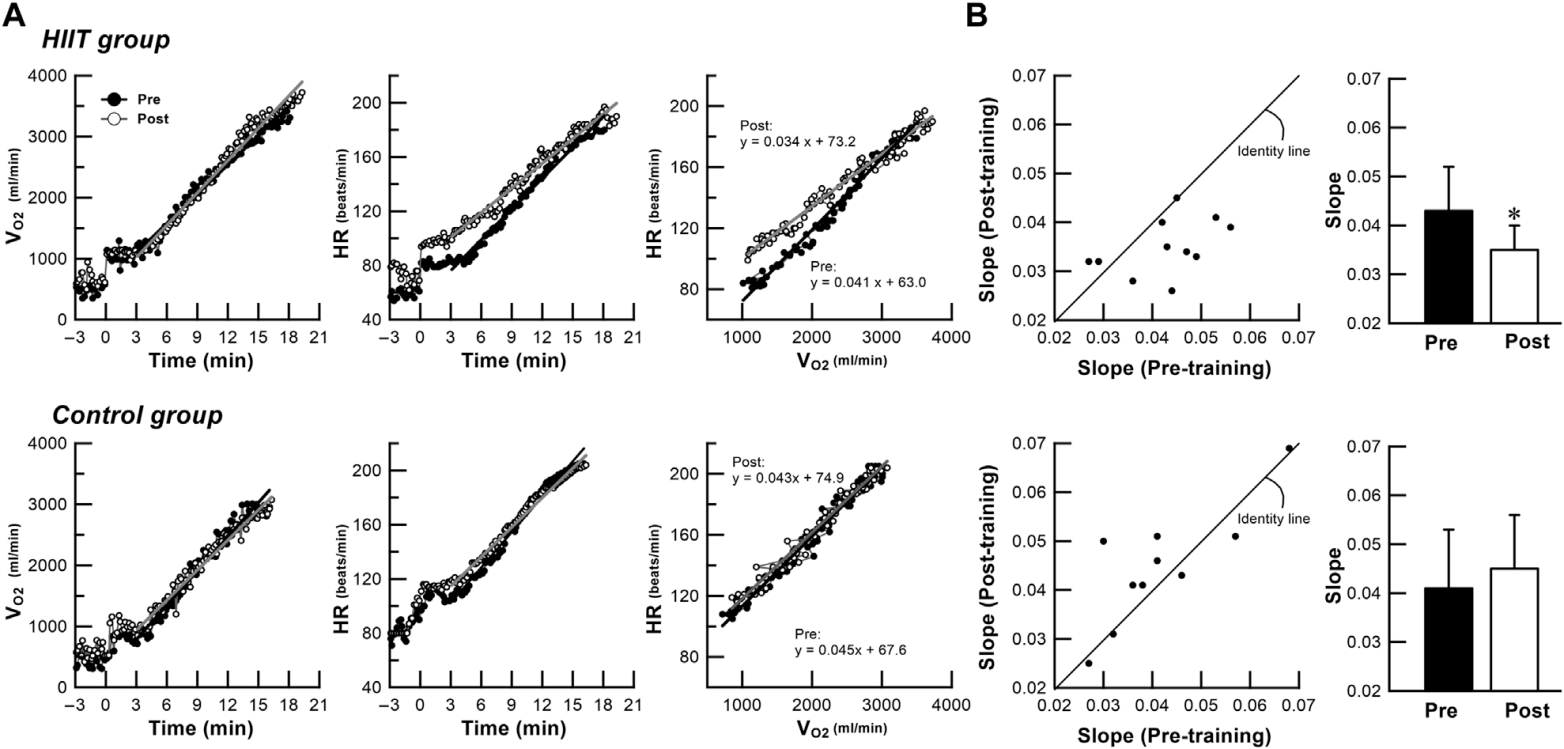
**(A)**: Data of a representative subject showing changes in V_O2_ and HR over time and V_O2_-HR relationship during ramp exercise test performed before and after intervention in the HIIT and control groups. **(B)**: Comparison of scatter- plots and bar charts of averaged data for slope of the V_O2_‒HR relationship during ramp exercise test before and after intervention in both groups. Close circles denote mean pre-intervention data. Open circles denote mean post-intervention data. Black bars denote mean pre-intervention data, and white bars denote mean post-intervention data. Vertical bars indicate ± SD. * *p* < 0.05 vs. pre-intervention value. In the trained representative subject, the intensity-dependent HR increase during ramp exercise test differs before and after the training, and is higher at low and middle intensity than at high intensity. However, no such changes are observed in the untrained representative subject **(A)**. The points below the identity line represent decreased measurement data in the post-training compared with pre-training in individual participants **(B)**. In the HIIT group, the slope of the V_O2_‒HR relationship decreases (*p* = 0.016). However, no significant differences in slope are found in the control group. HIIT high-intensity interval training, V_O2_ oxygen uptake, HR heart rate.

The time courses of V_O2_ and HR during the ramp exercise test and the maximum values of the cardiorespiratory variables and the maximum achievable work rate were compared before and after intervention in the HIIT and control groups (Figure 2A; Table 2). In the ramp exercise test, two-way ANOVA revealed a significant group × training interaction for V_O2max_ (+13%, *p* = 0.006), V_Emax_ (+27%, *p* < 0.001), RR_max_ (+13%, *p* = 0.003), HR_max_ (+3%, *p* = 0.046), SBP_max_ (+9%, *p* = 0.004) and WR_max_ (+13%, *p* < 0.001), indicating greater changes in these variables in the HIIT group than that in the control group. Analysis of simple interaction effect also confirmed that the changes were due to the significant training effects that improved those variables in the HIIT group.

Figure 2A shows the data of a representative subject in the HIIT group, the exercise intensity-dependent HR increase response during ramp exercise test was different before and after the HIIT program; HR response after the HITT program was higher than that before the program at low and middle intensities, but the two curves tended to converge at high intensity. However, no differences in exercise intensity-dependent HR response before and after intervention was observed in a representative subject in the control group, indicating that HIIT induced a faster HR response during dynamic exercise. This finding was supported by the V_O2_–HR relationship during ramp exercise test, which showed a significant decrease in the slope (*p* = 0.016) after intervention only in the HIIT group (Figure 2B).

The maximum values of the cardiorespiratory variables and the time to exhaustion during the step exercise test were compared before and after intervention in the HIIT and control groups (Table 2). In the step exercise test, two-way ANOVA revealed a significant group × training interaction and the main effect of training for V_Emax_ (+21%, *p* = 0.009), HR_max_ (+6%, *p* = 0.003), SBP_max_ (+11%, *p* = 0.001) and time to exhaustion (+95%, *p* < 0.001), indicating greater changes in these variables in the HIIT group than that in the control group. Analysis of simple interaction effect also confirmed that the changes were due to the significant training effects that improved those variables in the HIIT group.

**FIGURE 3:**
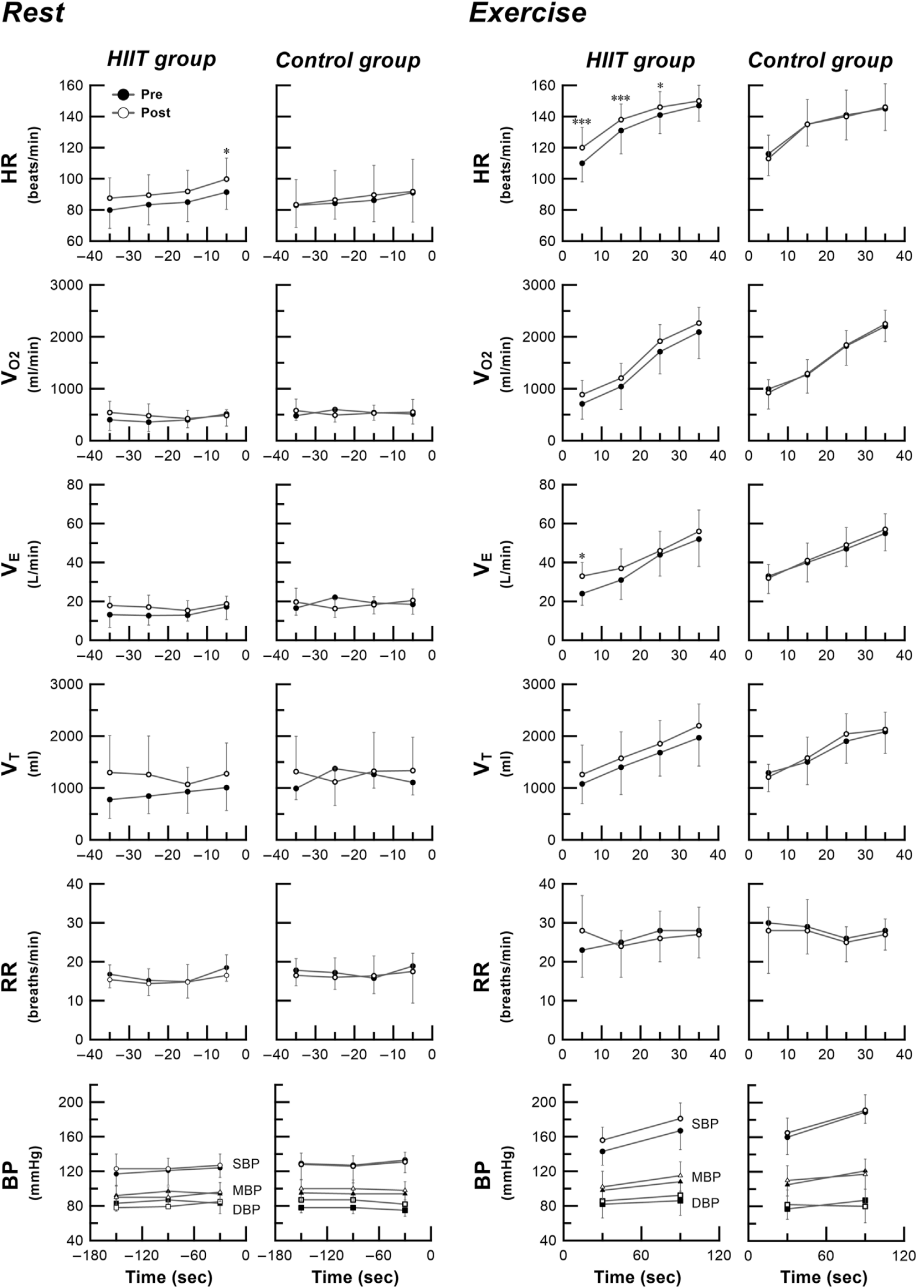
Time courses of cardiorespiratory responses preceding and after exercise during the step exercise test performed before and after intervention in the HIIT and control groups. Close circles denote mean pre-intervention data. Open circles denote mean post-intervention data. Vertical bars indicate ± SD. * *p* < 0.05, *** *p* < 0.001 vs. pre-intervention value. During the resting stage prior to exercise onset, time-dependent baseline shifts in HR are observed during step exercise test after training compared to before training in the HIIT group. Three-way ANOVA revealed a significant group × training interaction and main effect of training and time for HR during the resting period (-10 to 0 s, *p* = 0.020). However, no such changes are observed in the control group. After exercise onset, the changes in cardiorespiratory responses during step exercise test is higher after training compared to before training in the HIIT group. During the initial exercise periods, Three-way ANOVA revealed a significant group × training interaction and main effect of training and time for HR (0 to 10 s, *p* < 0.001; 10 to 20, *p* < 0.001; 20 to 30, *p* = 0.031) and V_E_ (0 to 10 s, *p* = 0.009). But no such change is observed in the control group. HIIT high-intensity interval training, HR heart rate, *V*_*O2*_ oxygen uptake, *V*_*E*_ minute ventilation, *V*_*T*_ tidal volume, *RR* respiratory rate, *BP* blood pressure, SBP systolic blood pressure, DBP diastolic blood pressure, MBP mean blood pressure.

Figure 3 shows the time courses of cardiorespiratory responses preceding and after exercise during the step exercise test performed before and after intervention in the HIIT and control groups. During the resting stage prior to exercise onset, time-dependent baseline shifts in HR are observed during step exercise test after training compared to before training in the HIIT group. Three-way ANOVA revealed a significant group × training interaction and main effect of training and time for HR during the resting period (−10 to 0 s, *p* = 0.020). However, no such changes are observed in the control group. However, no such changes are observed in the control group. After exercise onset, the changes in cardiorespiratory responses during step exercise test is higher after training compared to before training in the HIIT group. During the initial exercise periods, Three-way ANOVA revealed a significant group × training interaction and main effect of training and time for HR (0–10 s, *p* < 0.001; 10 to 20, *p* < 0.001; 20 to 30, *p* = 0.031) and V_E_ (0–10 s, *p* = 0.009). But no such change is observed in the control group.

**FIGURE 4:**
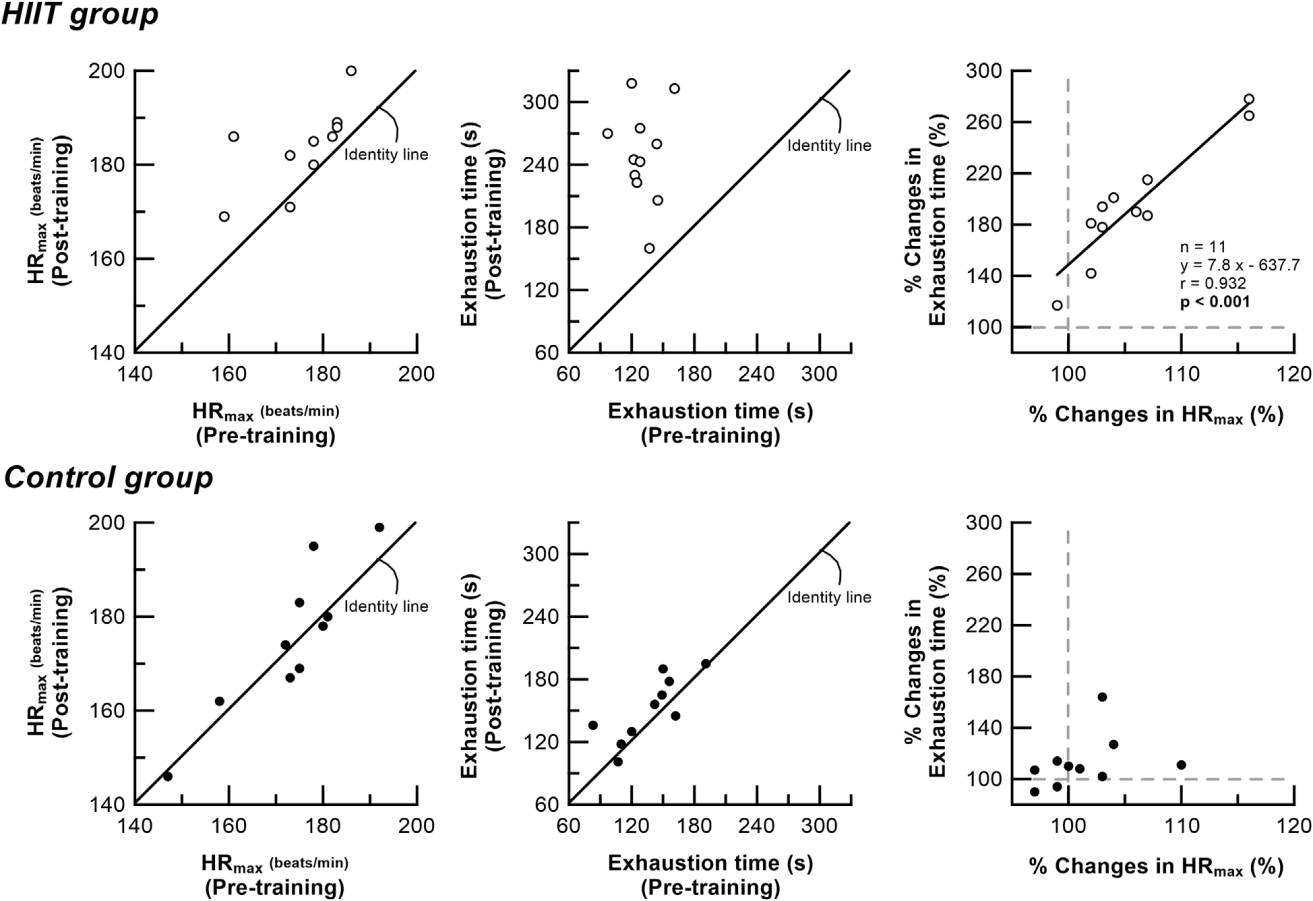
Relationship between HR_max_ and time to exhaustion in the step exercise test before and after intervention in the HIIT and control groups. Open circles denote individual data in the HIIT group. Close circles denote individual data in the control group. solid line is the linear regression line. In the HIIT group, there is no significant correlation between pre-intervention and post-intervention HR_max_ or time to exhaustion. However, there is a high correlation between percent change in time to exhaustion and percent change in HR_max_ (r = 0.932, *p* < 0.001) in the HIIT group. No correlation is found in the control group. *HR*_*max*_ maximal heart rate; Exhaustion time time to exhaustion during the step exercise.

Figure 4 shows the plots of pre- and post-intervention HR_max_ and time to exhaustion in the step exercise test. In the HIIT group, there was no significant correlation between pre- and post-intervention HR_max_, and between pre- and post-intervention time to exhaustion. However, there was a strong correlation between percent change (from before to after HIIT program) in time to exhaustion and percent change in HR_max_ (r = 0.932, *p* < 0.001). In the control group, no correlation was found in all the plots.

Table 3 compares the mean left ventricular morphological variables measured in resting supine position before and after intervention in the HIIT and control groups. Two-way ANOVA revealed a significant group × training interaction for posterior wall thickness (PWT) and left ventricular mass (LV mass), indicating greater changes in these variables in the HIIT group than those in the control group. Analysis of simple interaction effect also confirmed that the changes were due to the significant training effects on PWT and LV mass in the HIIT group. In the HIIT group, PWT and LV mass at rest increased by 11% (*p* = 0.001), 15% (*p* < 0.001), respectively, after the HIIT program compared to before the program. But no such change is observed in the control group.

**TABLE 3:** Comparison of resting left ventricular morphology before and after intervention in the HIIT and control groups.

	HIIT group		Control group		Mixed effect ANOVA (*p*-value)		
	(n = 11)		(n = 10)		Main effect		Interaction effect
	Pre	Post	Pre	Post	Group (G)	Training (T)	G × T
Rest							
LVEDd (cm)	5.2 ± 0.3	5.4 ± 0.3	5.1 ± 0.4	5.3 ± 0.4	0.351	0.006	0.316
LVESd (cm)	3.5 ± 0.3	3.5 ± 0.2	3.3 ± 0.4	3.4 ± 0.6	0.161	0.389	0.853
IVS (cm)	0.96 ± 0.13	0.99 ± 0.16	1.16 ± 0.1	1.13 ± 0.09	0.034	0.76	0.085
PWT (cm)	0.99 ± 0.13	1.09 ± 0.14**	1.22 ± 0.16	1.17 ± 0.1	0.016	0.06	<0.001
LV mass (g)	262 ± 52	303 ± 63***	301 ± 63	300 ± 68	0.825	<0.001	<0.001
Stroke volume (mL)	81 ± 18	91 ± 20	83 ± 17	85 ± 14	0.786	0.02	0.075

Values are presented as mean ± SD. HIIT, high-intensity interval training; LV, left ventricular; LVEDd, left ventricular end-diastolic dimension; LVESd, left ventricular end-systolic dimension; IVS, interventricular septal thickness; Pre, before intervention; Post, after intervention; PWT, posterior wall thickness. **p < 0.01, ***p < 0.001 vs. before HIIT, program.

## Discussion

### Effects of HIIT on maximal cardiorespiratory response during exercise

Many previous studies have shown that both endurance training and interval training generally increase V_O2max_ as an indicator of aerobic exercise capacity. In this study, we also demonstrated that once a week HIIT at 95% WR_max_ intensity improved not only V_O2max_ (+13% in ramp exercise test), but also the time to exhaustion in high-intensity step exercise test (+95%, *p* < 0.001) (Table 2). These results are consistent with our previous report that interval training at 80% WR_max_ intensity increased V_O2max_ by 13% and prolonged time to exhaustion during step exercise by 85% (Nakahara et al., 2015). Comparing our HIIT protocols at 95% WR_max_ and 80% WR_max_, the mean time to exhaustion during interval training was 2 min 10 s and 6 min 46 s, respectively. This result suggests that HIIT at 95% WR_max_ requires a relatively low contribution of aerobic energy metabolism during training than HIIT at 80% WR_max_. This may imply that substantial contribution of aerobic energy metabolism during training is not necessarily required to produce the effect of HIIT on maximal cardiorespiratory response. In a previous study, Matsuo et al. (2014) reported that an HIIT program of training 5 times a week at two different intensities (85%–90% vs. 120% V_O2max_) improved V_O2max_ by 23% and 17%, respectively. Stepto et al. (1999) also reported improved maximal power and 40-km time trial performance by two markedly different HIIT protocols (85% power output and 175% power output, twice a week). These results suggest that similar training effects can be obtained by HIIT regardless of training intensity. Interestingly, our study demonstrated experimentally that even at a training frequency of once weekly, HIIT exhibited cardiorespiratory effects (V_O2max_ increased by 8%–22% and V_Emax_ increased by 14%–25%), comparable to those reported for previous training studies using different exercise intensities, durations, and frequencies (Slørdahl et al., 2004; Roditis et al., 2007; Kim et al., 2016; Monks et al., 2017). Previous study has shown that HIIT increases V_O2max_ by inducing significant adaptive changes in the cardiovascular system (Helgerud et al., 2007). Indeed, in the present study, subjects who completed the HIIT program showed increases (from before to after HIIT program) in HR_max_ of 190–196 beats (+3%) and 174 to 184 beats (+6%) in ramp and step exercise tests, respectively; and increases in SBP_max_ of 189–206 mmHg (+9%) and 179–197 mmHg (+11%).

### Effects of HIIT on dynamic cardiorespiratory responses before and during ramp and step exercises

Another new finding in this study is that HIIT not only increases HR_max_ and SBP_max_ during maximal exercise, but also alters the time-dependent HR responses before and after the start of step exercise (Figure 3). There was a strong correlation between percent change (from before to after HIIT program) in time to exhaustion and percent change in HR_max_ during high-intensity exercise (Figure 4). After HIIT, the 10-s HR immediately before starting step exercise increased by 9% (from 91 bpm pre-HIIT to 100 bpm post-HIIT) and the HR increase was accelerated at the start of exercise and continued until almost the end of exercise. Recently, we demonstrated that the quantitative and temporal changes in cardiorespiratory responses during the pre-exercise resting anticipation period were dependent on the subsequent exercise intensity (0% WR_max_ vs. 95% WR_max_ task: from 72 bpm to 102 bpm, increased by 42%), suggesting a role of neural control mechanism in the higher brain (Miyamoto et al., 2022). The HIIT-induced time-dependent HR increase during pre-exercise anticipation period observed in this study supports the existence of exercise experience-based learning mechanism (Wood et al., 2003) that adequately explains the results of this study. Also, in a previous study, the time to exhaustion with and without preparation for HR responses from the exercise preparation period to just after initiation during high-intensity exercise changed by 15%. In addition, the enhanced exercise performance correlated positively with increased HR response just before and immediately after exercise onset (Miyamoto et al., 2022). In this study, despite the similar acceleration observed in both the HR during the preparatory period and the immediate post-exercise, which notably changed time to exhaustion (95%), no correlation was found between these factors. These findings suggest that the acceleration in circulatory response is not solely responsible for all aspects of exercise performance. Instead, they imply a complex influence of various adaptive changes in cardiorespiratory functions observed in this study, indicating that they collectively impact exercise performance. Other previous studies demonstrated that the time constant of cardiorespiratory response immediately after the start of moderate-intensity step exercise decreased significantly after HIIT and endurance training (Hagberg et al., 1980; McKay et al., 2009). These results indicate that adaptation to exercise training enables an individual to adjust more rapidly to the energy requirement of constant load submaximal work, resulting in a smaller O_2_ deficit. This is consistent with the present finding of an accelerated initial increase in V_E_ and HR response after exercise onset in subjects who completed the HIIT program (Figure 3). However, this study employed a high-intensity load (95% WR_max_) used in actual training sessions. Previous studies have indicated that the HR response before the initiation of high-intensity step exercise is altered in an intensity-dependent manner (Miyamoto et al., 2022). Considering these findings, relying on traditional low to moderate-intensity step loads in evaluating similar high-intensity training sessions might limit the assessment of specific training-induced adaptations before and after exercise initiation. Based on control system theory for a dynamic linear system (Whipp and Ward, 1980; Rossiter, 2011), the HIIT-induced change in slope of the V_O2_–HR relationship during the ramp exercise observed in this study indicates clearly that the HR response after the onset of exercise is accelerated, which leads to essentially the same conclusion as that obtained in the step exercise test. The decrease in resting BP due to exercise training has already been reported, and the results are consistent with previous studies (Tjønna et al., 2009; Dimeo et al., 2012; Domaradzki et al., 2022; Popowczak et al., 2022). Our novel finding that an HIIT program enhances time-dependent increase in HR response during exercise indicates that HIIT may increase sympathetic activation during all phases of high-intensity exercise from onset to end of exercise, resulting in functional enhancement of the central circulatory system during high-intensity exercise.

### Effects of HIIT on intense exercise performance and possible mechanisms

The mechanism of acceleration of HR response and marked improvement in intense exercise performance after once weekly HIIT for 8 weeks is unclear, but may be partly due to adaptive changes in autonomic circulatory regulation, which may greatly improve oxygen delivery at the onset of exercise. This may increase oxygen supply to muscles and decrease peripheral muscle fatigue, consequently contributing to improve intense exercise performance (Sietsema et al., 1989; Amann and Calbet, 2008). We have previously shown that HR increase prior to and after the onset of step exercise under anticipation conditions is strongly associated with time to exhaustion (Miyamoto et al., 2022). Therefore, the observation of a significant increase in HR from pre-exercise resting period in the HIIT group suggests that anticipatory cardiorespiratory control (feedforward control) is also affected by HIIT, which may partly explain the improvement in intense exercise performance after training. On the other hand, the finding that the improvement in intense exercise performance was more pronounced in the step exercise test than in the ramp exercise test (step: +95% time to exhaustion vs. ramp: +13% WR_max_) seems to imply that improvement of exercise performance is determined not only by maximum aerobic capacity of the cardiorespiratory system, but also by various physiological mechanisms including changes in anaerobic capacity in the body.

Although few previous studies have investigated changes in maximal HR and SBP and dynamic HR responses to exercise after HIIT, some reports showed that HR_max_ was unchanged (Warburton et al., 2004; Roditis et al., 2007; Nakahara et al., 2015) or decreased (−4%) (Monks et al., 2017), and SBP_max_ did not change after HIIT training (Warburton et al., 2004). Our previous HIIT study using 80% WR_max_ intensity also detected no difference in HR_max_ during ramp exercise (Nakahara et al., 2015). The reason for the difference between the present findings and previous results is unclear, but may be related to the difference in exercise intensity of HIIT and/or the difference in training method to reach exhaustion.

### Effect of HIIT on left ventricular morphology

In this study, significant increases in PWT (+12%), LV mass (+12%) were observed after undergoing HIIT at 95% WR_max_ once a week for 8 weeks. Our previous HIIT study also showed similar results (Nakahara et al., 2015). Our previous study performed at 80% WR_max_ once a week for 12 weeks achieved comparable training effects (PWT: +18%, LV mass: +28%) (Nakahara et al., 2015), and morphological changes of the left ventricle were also observed. Haykowsky et al. (1998) observed that short-term high-intensity endurance training for 3 weeks induced rapidly increasing adaptation only for PWT (+7%), but not for LV mass or left ventricular end-diastolic dimension (LVEDd). In addition, Matsuo et al. (2014) compared the effects of different intensities (85%–90% vs. 120% V_O2max_) of HIIT (5 times a week for 8 weeks) and observed similar increase in LV mass (+8% and +6%, respectively) regardless of training intensity. The findings of these previous studies are generally consistent with the results of this study. On the other hand, Matsuo et al. (2014) reported a decrease in resting HR (−5 to −9%) and increases in LVEDd (+8–10%) and stroke volume (SV) (+4–9%) after HIIT. Helgerud et al. (2007) also showed an increase in SV (+9–10%) during maximal exercise after HIIT. On the contrary, we found no significant changes in resting HR, LVEDd and SV after HIIT, both in the present and previous studies (Nakahara et al., 2015).

Physiological adaptive changes in the heart of athletes have been found to vary depending on training modalities (Morganroth et al., 1975; Adams et al., 1981; Zeppilli et al., 1995). Morganroth et al. (1975) reported that increased LV mass in long distance runners and swimmers undergoing endurance training was associated with extensional hypertrophy by increasing LVEDd, and that increased LV mass in wrestlers undergoing interval and resistance training was associated with concentric hypertrophy by increasing ventricular septal thickness (IVS) and PWT. In addition, Fagard et al. (1983) reported that training-associated increases in IVS and PWT were due to concentric left ventricular hypertrophy resulting from a rapid increase in systolic blood pressure. In this study, the significant increase in SBP during ramp and step exercise tests after HIIT indicated that the stress load on the heart was sufficient, and the subjects exercised until exhaustion during each training session. These results suggest that the increases in PWT and LV mass are due to concentric left ventricular hypertrophy caused by repeated stress to the heart. The reason for the difference in training effect among studies is not clear, but may depend on the nature of the training program, such as the frequency, volume and method of training.

## Conclusion

We have demonstrated that HIIT at 95% WR_max_ induces training-specific adaptations such as improved cardiorespiratory adjustments, not only during maximal exercise but also before and after the onset of high-intensity exercise, improvement of exercise performance mainly associated with circulatory systems accompanied by left ventricular hypertrophy, which are comparable to the effects of less intense (80% WR_max_) HIIT reported previously. Furthermore, the comparison with the control group provided additional evidence of the efficacy of HIIT at 95% WR_max_ in inducing these advantageous adaptations.

## Study limitation

Age and gender are well known to have significant impact on cardiac response to exhaustive upright cycle exercise. For example, previous studies indicate differential regulation of cardiovascular function and exercise efficiency between sexes during constant-load submaximal exercise (Fleg et al., 1995; Charkoudian and Joyner, 2004; Wheatley et al., 2014). Therefore, female and older participants were not included in this study.

Regarding the sample size, ideally, the number of participants, including women, should have been increased to at least two digits for a more robust validation. However, due to the challenges of conducting a training experiment with such a large number of indicators and the limitations of time and physical resources to measure multiple indicators, it was not possible to gather the desired number of participants during the planning stage of this experiment. In the future, it is crucial to devise strategies to increase the sample size and conduct experiments effectively.

The training effect is strongly influenced by the subjects’ initial fitness level and training status. In our studies of the effects of once weekly HIIT so far, the subjects were healthy male college students with normal fitness, but not well-trained athletes. It is possible that training effects are acquired easily in these subjects. However, it remains unclear whether HIIT will produce similar effects in well-trained athletes. Moreover, participants in this study required substantial motivation during the training period. Therefore, less motivated subjects, especially persons with low fitness level or patients with various diseases, may have difficulties in performing the severe-intensity interval training. Further study is thus needed to design training programs with intensity and frequency appropriate for persons with low fitness level or patients with various diseases, and examine the effects in these study populations.

To address concerns about the small size of our participant group and determine an appropriate sample size, we conducted a comprehensive power analysis. This analysis considered the variability in effect sizes across various measures, employing a conservative approach to estimate these sizes based on outcomes observed in comparable studies. With a significance level (α) set at 0.05 and a target statistical power of 0.80, our analysis revealed that the optimal sample size is not fixed but varies depending on the specific effect sizes under consideration. Initially, the study began with a sample size of 21 participants, encompassing both the HIIT group and the control group. However, in-depth analyses, we observed a range of effect sizes, ultimately indicating that the optimal sample size should ideally range from 24 to 40 participants to attain a sufficient level of statistical power. It should be noted that despite our efforts to augment the sample size, the inherent challenges associated with conducting a training experiment posed substantial difficulties, preventing us from achieving the desired increase in sample size. Furthermore, it is essential to recognize that all examined indicators consistently revealed a substantial interaction effect between the group and training factors, underscoring the significance of this finding despite the constraints imposed by the sample size. In future research endeavors, it may prove advantageous to contemplate a larger sample size to strengthen the robustness of the results and enhance the generalizability of the findings. Additionally, exploring potential additional factors that could exert an influence on the observed effects may contribute to a more comprehensive understanding of the phenomena under investigation.

## Data Availability

The original contributions presented in the study are included in the article/supplementary material, further inquiries can be directed to the corresponding author.

## References

[B1] Adams T. D. Yanowitz F. G. Fisher A. G. Ridges J. D. Lovell K. Pryor T. A. (1981). Noninvasive evaluation of exercise training in college-age men. Circulation 64, 958–965. 10.1161/01.cir.64.5.958 7285309

[B2] Amann M. Calbet J. A. L. (2008). Convective oxygen transport and fatigue. J. Appl. Physiol. 104, 861–870. 10.1152/japplphysiol.01008.2007 17962570

[B3] Charkoudian N. Joyner M. J. (2004). Physiologic considerations for exercise performance in women. Clin. Chest Med. 25, 247–255. 10.1016/j.ccm.2004.01.001 15099886

[B4] Devereux R. B. Alonso D. R. Lutas E. M. Gottlieb G. J. Campo E. Sachs I. (1986). Echocardiographic assessment of left ventricular hypertrophy: comparison to necropsy findings. Am. J. Cardiol. 57, 450–458. 10.1016/0002-9149(86)90771-X 2936235

[B5] Dimeo F. Pagonas N. Seibert F. Arndt R. Zidek W. Westhoff T. H. (2012). Aerobic exercise reduces blood pressure in resistant hypertension. Hypertension 60, 653–658. 10.1161/HYPERTENSIONAHA.112.197780 22802220

[B6] Domaradzki J. Koźlenia D. Popowczak M. (2022). Prevalence of positive effects on body fat percentage, cardiovascular parameters, and cardiorespiratory fitness after 10-week high-intensity interval training in adolescents. Biol. (Basel) 11, 424–515. 10.3390/biology11030424 PMC8945095 35336798

[B7] Fagard R. Aubert A. Lysens R. Staessen J. Vanhees L. Amery A. (1983). Noninvasive assessment of seasonal variations in cardiac structure and function in cyclists. Circulation 67, 896–901. 10.1161/01.CIR.67.4.896 6825246

[B8] Fleg J. L. O’Connor F. Gerstenblith G. Becker L. C. Clulow J. Schulman S. P. (1995). Impact of age on the cardiovascular response to dynamic upright exercise in healthy men and women. J. Appl. Physiol. 78, 890–900. 10.1152/jappl.1995.78.3.890 7775334

[B9] Hagberg J. M. Hickson R. C. Ehsani A. A. Holloszy J. O. (1980). Faster adjustment to and recovery from submaximal exercise in the trained state. J. Appl. Physiol. Respir. Environ. Exerc. Physiol. 48, 218–224. 10.1152/jappl.1980.48.2.218 7364606

[B10] Haykowsky M. J. Smith D. J. Malley L. Norris S. R. Smith E. R. (1998). Effects of short-term altitude training and tapering on left ventricular morphology in elite swimmers. Can. J. Cardiol. 14, 678–681. 9627523

[B11] Helgerud J. Høydal K. Wang E. Karlsen T. Berg P. Bjerkaas M. (2007). Aerobic high-intensity intervals improve VO2max more than moderate training. Med. Sci. Sports Exerc. 39, 665–671. 10.1249/mss.0b013e3180304570 17414804

[B12] Kercher V. M. Kercher K. Levy P. Bennion T. Alexander C. Amaral P. C. (2023). 2023 fitness trends from around the globe. ACSM’s Heal. Fit. J. 27, 19–30. 10.1249/FIT.0000000000000836

[B13] Kim C. H. Wheatley C. M. Behnia M. Johnson B. D. (2016). The effect of aging on relationships between lean body mass and VO2max in rowers. PLoS One 11, 01602755–e160311. 10.1371/journal.pone.0160275 PMC4968829 27479009

[B14] Litleskare S. Enoksen E. Sandvei M. Støen L. Stensrud T. Johansen E. (2020). Sprint interval running and continuous running produce training specific adaptations, despite a similar improvement of aerobic endurance capacity—a randomized trial of healthy adults. Int. J. Environ. Res. Public Health 17, 3865. 10.3390/ijerph17113865 32485945 PMC7312918

[B15] Matsuo T. Saotome K. Seino S. Shimojo N. Matsushita A. Iemitsu M. (2014). Effects of a low-volume aerobic-type interval exercise on VO2max and cardiac mass. Med. Sci. Sports Exerc. 46, 42–50. 10.1249/MSS.0b013e3182a38da8 23846165

[B16] McKay B. R. Paterson D. H. Kowalchuk J. M. (2009). Effect of short-term high-intensity interval training vs continuous training on O 2 uptake kinetics, muscle deoxygenation, and exercise performance. J. Appl. Physiol. 107, 128–138. 10.1152/japplphysiol.90828.2008 19443744

[B17] Miyamoto T. Sotobayashi D. Ito G. Kawai E. Nakahara H. Ueda S. (2022). Physiological role of anticipatory cardiorespiratory responses to exercise. Physiol. Rep. 10, e15210–e15232. 10.14814/phy2.15210 35246949 PMC8897741

[B18] Monks L. Seo M. W. Kim H. B. Jung H. C. Song J. K. (2017). High-intensity interval training and athletic performance in Taekwondo athletes. J. Sports Med. Phys. Fit. 57, 1252–1260. 10.23736/S0022-4707.17.06853-0 28085127

[B19] Morganroth J. Maron B. J. Henry W. L. Epstein S. E. (1975). Comparative left ventricular dimensions in trained athletes. Ann. Intern. Med. 82, 521–524. 10.7326/0003-4819-82-4-521 1119766

[B20] Nakahara H. Ueda S. Y. Miyamoto T. (2015). Low-frequency severe-intensity interval training improves cardiorespiratory functions. Med. Sci. Sports Exerc. 47, 789–798. 10.1249/MSS.0000000000000477 25137370

[B21] Nakahara H. Ueda S. Y. Miyamoto T. (2020). Low frequency severe-intensity interval training markedly alters respiratory compensation point during incremental exercise in untrained male. Front. Physiol. 11, 1100–1108. 10.3389/fphys.2020.01100 33013469 PMC7498695

[B22] Popowczak M. Rokita A. Koźlenia D. Domaradzki J. (2022). The high-intensity interval training introduced in physical education lessons decrease systole in high blood pressure adolescents. Sci. Rep. 12, 1974–1977. 10.1038/s41598-022-06017-w 35132123 PMC8821617

[B23] Roditis P. Dimopoulos S. Sakellariou D. Sarafoglou S. Kaldara E. Venetsanakos J. (2007). The effects of exercise training on the kinetics of oxygen uptake in patients with chronic heart failure. Eur. J. Cardiovasc. Prev. Rehabil. 14, 304–311. 10.1097/hjr.0b013e32808621a3 17446812

[B24] Rognmo Ø. Hetland E. Helgerud J. Hoff J. Slørdahl S. A. (2004). High intensity aerobic interval exercise is superior to moderate intensity exercise for increasing aerobic capacity in patients with coronary artery disease. Eur. J. Cardiovasc. Prev. Rehabil. 11, 216–222. 10.1097/01.hjr.0000131677.96762.0c 15179103

[B25] Rossiter H. B. (2011). Exercise: kinetic considerations for gas exchange. Compr. Physiol. 1, 203–244. 10.1002/cphy.c090010 23737170

[B26] Sahn D. J. DeMaria A. Kisslo J. Weyman A. (1978). Recommendations regarding quantitation in M-mode echocardiography: results of a survey of echocardiographic measurements. Circulation 58, 1072–1083. 10.1161/01.CIR.58.6.1072 709763

[B27] Seiler S. Hetlelid K. J. (2005). The impact of rest duration on work intensity and RPE during interval training. Med. Sci. Sports Exerc. 37, 1601–1607. 10.1249/01.mss.0000177560.18014.d8 16177614

[B28] Sietsema K. E. Daly J. A. Wasserman K. (1989). Early dynamics of O2 uptake and heart rate as affected by exercise work rate. J. Appl. Physiol. 67, 2535–2541. 10.1152/jappl.1989.67.6.2535 2606862

[B29] Slørdahl S. A. Madslien V. O. E. Støylen A. Kjos A. Helgerud J. Wisløff U. (2004). Atrioventricular plane displacement in untrained and trained females. Med. Sci. Sports Exerc. 36, 1871–1875. 10.1249/01.MSS.0000145444.01292.3D 15514500

[B30] Stepto N. K. Hawley J. A. Dennis S. C. Hopkins W. G. (1999). Effects of different interval-training programs on cycling time-trial performance. Med. Sci. Sports Exerc. 31, 736–741. 10.1097/00005768-199905000-00018 10331896

[B31] Tabata I. Nishimura K. Kouzaki M. Hirai Y. Ogita F. Miyachi M. (1996). Effects of moderate-intensity endurance and high-intensity intermittent training on anaerobic capacity and VO(2max). Med. Sci. Sports Exerc. 28, 1327–1330. 10.1097/00005768-199610000-00018 8897392

[B32] Tjønna A. E. Stølen T. O. Bye A. Volden M. Slördahl S. A. Ødegård R. (2009). Aerobic interval training reduces cardiovascular risk factors more than a multitreatment approach in overweight adolescents. Clin. Sci. 116, 317–326. 10.1042/CS20080249 18673303

[B33] Warburton D. E. R. Haykowsky M. J. Quinney H. A. Blackmore D. Teo K. K. Taylor D. A. (2004). Blood volume expansion and cardiorespiratory function: effects of training modality. Med. Sci. Sports Exerc. 36, 991–1000. 10.1249/01.MSS.0000128163.88298.CB 15179169

[B34] Wheatley C. M. Snyder E. M. Johnson B. D. Olson T. P. (2014). Sex differences in cardiovascular function during submaximal exercise in humans. Springerplus 3, 445–513. 10.1186/2193-1801-3-445 25191635 PMC4153874

[B35] Whipp B. Ward S. (1980). Ventilatory control dynamics during muscular exercise in man. Int. J. Sports Med. 01, 146–159. 10.1055/s-2008-1034653

[B36] Wisløff U. Støylen A. Loennechen J. P. Bruvold M. Rognmo Ø. Haram P. M. (2007). Superior cardiovascular effect of aerobic interval training versus moderate continuous training in heart failure patients: a randomized study. Circulation 115, 3086–3094. 10.1161/CIRCULATIONAHA.106.675041 17548726

[B37] Wood H. E. Fatemian M. Robbins P. A. (2003). A learned component of the ventilatory response to exercise in man. J. Physiol. 553, 967–974. 10.1113/jphysiol.2003.047597 14514870 PMC2343621

[B38] Zeppilli P. Vannicelli R. Santini C. Russo A. Picani C. Pulmieri V. (1995). Echocardiographic size of conductance vessels in athletes and sedentary people. Int. J. Sports Med. 16, 38–44. 10.1055/s-2007-972961 7713629

